# A Node Linkage Approach for Sequential Pattern Mining

**DOI:** 10.1371/journal.pone.0095418

**Published:** 2014-06-16

**Authors:** Osvaldo Navarro, René Cumplido, Luis Villaseñor-Pineda, Claudia Feregrino-Uribe, Jesús Ariel Carrasco-Ochoa

**Affiliations:** Departamento de Ciencias Computacionales, Instituto Nacional de Astrofísica, Óptica y Electrónica, Sta. Ma. Tonantzintla, Puebla, México; University of Catania, Italy

## Abstract

Sequential Pattern Mining is a widely addressed problem in data mining, with applications such as analyzing Web usage, examining purchase behavior, and text mining, among others. Nevertheless, with the dramatic increase in data volume, the current approaches prove inefficient when dealing with large input datasets, a large number of different symbols and low minimum supports. In this paper, we propose a new sequential pattern mining algorithm, which follows a pattern-growth scheme to discover sequential patterns. Unlike most pattern growth algorithms, our approach does not build a data structure to represent the input dataset, but instead accesses the required sequences through pseudo-projection databases, achieving better runtime and reducing memory requirements. Our algorithm traverses the search space in a depth-first fashion and only preserves in memory a pattern node linkage and the pseudo-projections required for the branch being explored at the time. Experimental results show that our new approach, the Node Linkage Depth-First Traversal algorithm (NLDFT), has better performance and scalability in comparison with state of the art algorithms.

## Introduction

Since Agrawal [1] proposed the problem of sequential pattern mining, it has become an important data mining problem, mainly because of its wide variety of applications. Sequential pattern mining methods have been used in applications such as mining web usage behaviour [2], [3], [4], Drug-drug interaction detection [5], text mining tasks such as document clustering [6], question answering [7], authorship atribution [8], touring path suggestion [9], CRM strategies for online shopping [10], mining anomalous events in surveillance videos from commercial environments [11], among others. This range of applications is possible likely because datasets from many domains contain some sort of sequential order between its elements; for instance, the sequential order of proteins in DNA sequences, time stamps on web access logs and business databases, the sequence of words in a document, among others. Furthermore, due to the dramatic pace at which data is collected nowadays, most of the current data mining methods are becoming inefficient. This is because most of the popular data mining methods were created when the common dataset size was several orders of magnitude smaller [12].

Thus, current datasets pose a significant challenge for current data mining approaches, which is part of the motivation for this work.

The two main approaches to tackle the sequential mining problem are: apriori-based and pattern growth-based [13]. The first strategy consists in generating a set of candidate patterns and then testing them against the frequency threshold to find the sequential patterns. Pruning techniques are usually combined with this strategy, to generate a candidate pattern set as small as possible. However, approaches based on this strategy have to deal with a combinatorial explosion of candidate patterns when dealing with large datasets and low frequency thresholds. The pattern-growth strategy finds sequential patterns by appending symbols to an already know frequent sequence, forming new sequences, which then are evaluated to see if they are frequent as well. The pattern-growth based approaches usually do this by traversing a previously build data structure that represents the entire dataset. This data structures become huge when processing large databases, which usually has a negative impact on performance and memory usage.

In this paper, the Node Linkage Depth-First Traversal (NLDFT) algorithm for sequential pattern mining is proposed. Unlike most pattern growth approaches, the proposed algorithm does not build a data structure to represent the input dataset, but instead accesses the required sequences through pseudo-projection databases. The proposed algorithm traverses the search space in a depth-first fashion and only preserves in memory a pattern node linkage and the pseudo-projections required for the branch being explored at the time.

The rest of this paper is organized as follows. The *Related Work* section presents a summary of the related work about sequential pattern mining. The Preliminaries section presents the basic concepts and ideas necessary to understand the sequential pattern mining problem and its formal definition. In the *Proposed Algorithm* section, the NLDFT algorithm is described. The *Performance Evaluation* section describes the experiments made to evaluate the proposed algorithm and the results obtained from them. Next, the Complexity Analysis section presents a theoretical analysis and comparison of the performance of the NLDFT algorithm. Finally, the Conclusions* and Future Work* section presents general conclusions and suggestions for future work.

### Related Work

There is a variety of problems that deal with mining frequent sequences, each one with different input and/or conditions. Among them, for instance, there is the problem of finding frequent sequences of symbols [1], frequent sequences of itemsets [14], maximal frequent word sequences [15], frequent closed sequences [16], frequent sequences with time constraints [17], among others. In this work we addressed the problem of mining frequent sequences of symbols, which will be referred to as *sequential pattern mining* in the rest of this article.

The sequential pattern mining problem was first defined in [1]. Since then, many other approaches have been proposed. These methods can be categorized, by the way they discover sequential patterns, in three categories: apriori-based approaches, pattern growth-based approaches and hybrid approaches.

The apriori-based approaches usually discover the sequential patterns in a breadth-first fashion, *i.e.*, they obtain the frequent sequences of size *k* together at the *k* iteration of the algorithm as it traverses the search space. Also, these methods depend on a feature named *generate-and-test* [18] to carry out the mining. This feature entails growing already found patterns by one item at a time and then testing the generated candidates against the minimum support.

The first algorithm that worked under the apriori-based scheme was AprioriAll, which was proposed by Agrawal [1]. The AprioriAll algorithm was proposed to tackle the problem of finding sequential patterns in a database of customer transactions. This algorithm travels the search space in a first-breadth fashion, finding all the patterns of length *k* in the *k* iteration before moving on to the next one. To find the patterns in an iteration level, this method generates a set of candidate patterns, which are further tested against the minimum support. Also, the algorithm incorporates a pruning technique based on the antimonotonic property that states that if a sequence cannot pass the minimum support test, all of its super-sequences will also fail the test [18]. This method has the main disadvantage of generating an explosive number of candidates, particularly at early stages of the mining process, which consumes a lot of memory.

Another representative algorithm of the apriori-based scheme is SPADE [19]. This method not only discovers single item sequences, but sequences of subsets of items, as well. SPADE represents the search space as a lattice of frequent sequences, which can be traversed in either depth-first or breadth-first fashion. The algorithm traverses the lattice, testing candidates against the minimum support to find the set of frequent sequences. To achieve this, an id-list is generated for each frequent sequence in the lattice, which is a list of all the input-sequence identifiers and item set identifier pairs that contain that sequence. To obtain the support count of a candidate sequence *k*-pattern, the algorithm performs temporal joins over the id-lists of any two of its (*k*−1) subsequences. Moreover, as maintaining all the intermediate id-list in memory would not be possible for databases of considerable size, Zaki proposed to break the lattice into disjoint subsets called *equivalence classes*. Thus, each equivalence class can be loaded into main memory and processed separately. However, even with the improvement of not having to constantly read the original database, but only three times, this method still faces the problem of generating a huge number of candidates, specially when dealing with large databases and/or low minimum supports.

To tackle the issue of efficiently generating candidate patterns, Tan et al. [20] proposed *SEQUEST*, an algorithm that relies on a structure called Direct Memory Access Strips (DMA-Strips). A DMA-Strip represents a single sequence in a database, and is composed by an ordered list which stores a sequence of items' *label*, *scope* and *event-id*. The *event-id* groups items based on their timestamps and the *scope* is used to determine the relationship between two consecutive items in a strip. To generate a candidate pattern, the algorithm iterates through a DMA-Strim and extends one item at a time. Next, to test a candidate pattern against the minimum support, two approaches were proposed: a vertical join counting approach similar to the one used in [19] and a horizontal counting approach using a hash table. Experimental results indicated that the vertical approach performed better than the horizontal approach. However, the vertical approach performance could degrade if the frequency of extracted candidate patterns is high, as it usually occurs when the minimum support is low, due to the nature of the join approach complexity.

A recent apriori-based approach is *PRISM* [14]. In this approach, an encoding based on prime factorization theory is used to represent the input database, in order to efficiently calculate the frequency of candidate patterns. PRISM represents the search space as a tree structure, where each node represents a sequence, and generates candidate patterns by combining a node with each one of its siblings. Finally, PRISM uses join operations over the encoded database to find the frequency of the current candidate. Although PRISM outperforms previous apriori-based approached such as SPADE, it also has the issue of the combinatorial explosion of the candidate generation process, which would significantly increase the runtime of the algorithm when dealing with large databases and low minimum supports.

The pattern growth strategy is characterized by traversing the search space in a depth-first fashion. It offers the advantage of not having to generate candidate patterns, which avoids the combinatorial explosion of patterns when dealing with large databases. Because of this, the pattern growth strategy has become the most used scheme of action, preferred over the apriori-based strategy. Among these approaches, Ezeife and Lu [2] presents the algorithm *Pre-order Linked Wap-Tree (PLWAP)*, a method for mining sequential patterns from a web access sequence database, which uses a data structure named *PLWAP-tree* [21] to efficiently access the sequences to be mined. The algorithm then recursively mines the tree, using prefix conditional sequence search, to find all the sequential patterns. One of the benefits of this method is that it only reads the original database twice, which helps to reduce I/O access costs or extra storage requirements. Also, the algorithm doesn't have to build a data structure at each level of recursion, which yields an improvement in running time, compared with previous works, which used the same data structure [3]. Peterson and Tang [22] propose an algorithm which also uses a data structure based on the aggregate tree. However, this method does not link the elements of the same type in the aggregate tree, but instead builds a forest of *first-occurrence subtrees* as the basic data structure to represent database projections. Although this method improves in memory usage compared with the PLWAP algorithm, it still has to build the entire aggregate tree before the mining process. This would require a lot of memory when dealing with large databases, which is usually the case within the text mining area. Ezeife et al. [23] presents PLWAPLong, also based on the PLWAP algorithm. PLWAPLong uses a simpler numbering scheme than PLWAP, to identify ancestor and descendant relationships in the aggregate tree. In this way, PLWAPLong is capable of dealing with larger databases than PLWAP, with a lower impact in performance. Moreover, PLWAPLong uses a more efficient approach to find the first occurrences of a word in the aggregate tree, which is a recurrent operation in the mining process, used to determine the frequency of a word in a projected database. While PLWAPLong can process larger databases than PLWAP, the algorithm was tested against databases with rather small vocabularies and average document size, in comparison with the ones used in text mining tasks. Furthermore, PLWAPLong too has to build the entire aggregate tree before the mining process, which results costly when dealing with databases with large vocabularies and a large average document size.

The hybrid approaches comprehend methods that combine one or more features from both apriori-based and pattern growth-based approaches. Among them, Chen [13] proposes a novel data structure named *UpDown Directed Acyclic Graph* (UDDAG), which supports bidirectional pattern growth from both ends of the discovered patterns, in order to perform a fast conditional search. First, the algorithm finds the frequent words in the sequence database, and uses them to filter the database from infrequent elements. Next, a frequent word is chosen to be the root of an UDDAG, and two database projections are created; one projection is formed by the prefixes of the sequence database with respect to the root pattern and the other one is formed by the suffixes. Frequent words are then found in these projections, and are appended to the root pattern to form new frequent patterns; thus it is said that in each level of recursion the root pattern is grown from both ends. Finally, the algorithm combines the patterns found in both projections to form candidate patterns, which are tested against the minimum support, in order to find new frequent patterns. Each one of the projections created is treated as a new database and the previous process is repeated, recursively. The UDDAG algorithm is an hybrid approach, as it uses conditional search in combination with candidate generation, to find all the sequential patterns. One of the advantages of its novel data structure, is that in each level or recursion the root pattern is grown from both sides concurrently, in comparison with other pattern growth-based methods, that only grow the length of the root pattern in 1 at each recursion. However, the need to use a candidate generation phase at each level of recursion impacts negatively in the algorithm's performance, and this impact becomes unacceptable when processing large databases using low minimum supports, due to the explosive number of candidates generated.

Many approaches have been proposed to provide an efficient solution to sequential pattern mining problem. However, the current methods still have problems when dealing with large databases and low minimum supports, which is the case in areas like Text Mining, where the datasets are usually formed by a large number of documents of considerable size. Two of the most common issues that occur when processing a large dataset are the combinatorial explosion of candidates in apriori-based approaches, and the generation of huge data structures in pattern growth-based approaches. The proposed algorithm avoids the candidate generation phase, and does not build a data structure to represent the input database, but only access the required sequences through pseudo-projected databases, and only maintains in memory these pseudo-projections along with a pattern-node linkage required in the branch of the search space being explored at a time, thus avoiding the common issues previously mentioned.

### Preliminaries

Before giving the formal definition of the sequential pattern mining problem, it is necessary to introduce the following definitions:

**Definition 1.** *Let* 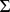 *be the set of symbols and ‘*  *’ be the operation of concatenating any two symbols from* 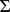 *. A* **non-empty sequence** *s is a finite succession of symbols from* 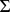 *,* 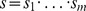 *, such that* 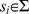 *for all* 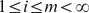 *and* 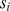 *and* 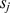 *are not necessarily different for* 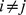 *. The length of a sequence* 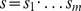 *is m, and it will denoted as* 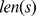 *. A* **sequence database** *D is a finite set of non-empty sequences. A* **pattern** *is a non-empty sequence.*

**Definition 2.** *A non-empty sequence is a* **subsequence** *of another sequence if it is embedded in that sequence, i.e., a sequence* 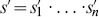 *is a subsequence of a sequence* 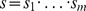 *, denoted as* 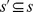 *, if and only if* 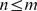 *and there exist* 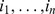 *such that* 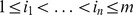 *and* 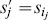 *for all* 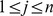 *.*

**Definition 3.** *A sequence s in D is said to support a pattern p if p is a subsequence of s. The* **support of a pattern** *p in D, denoted as* 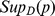 *, is the number of sequences in D that support p. Given a threshold ξ in* 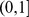 *, a pattern p is frequent with respect to ξ and D if* 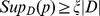 *, where* 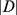 *is the number of sequences in D.* 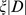 *is called the absolute threshold and denoted as η.*

**Definition 4.** *Given two sequences s and p, such that* 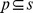 *, the* **p-prefix** *of s is the prefix of s from the first symbol (the leftmost symbol) to the first occurrence of p inclusive. This definition is a generalization of the one found in* [*24*] *, for sequences of length greater than 1.*

For instance, considering 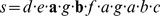 and 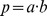, the p-prefix of *s* is 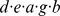. The subsequence 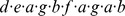 is not the p-prefix because the first occurrence of 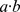 in the sequence is the one that is in bold.

**Definition 5.** *Given two sequences s and p, such that* 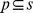 *, s can be represented as* 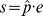 *, where* 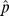 *is the p-prefix of s and e is the rest of the sequence. The subsequence e corresponds to the* **projection** *of s with respect to p, denoted as proj(s,p).*

*Roughly speaking, the projection of a sequence s with respect to a sequence p can be constructed by taking out the symbols in s, from the first one to the last symbol in the first occurrence of p in s. The remaining symbols will constitute the projection. For instance, considering* 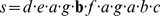 *and* 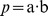 *, the projection of s with respect to p is* 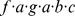 *.*

**Definition 6.** *Given a sequence database D and a sequence s, the* **projection database** *of D with respect to s, denoted as* 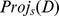 *is defined as the set of non-empty projections of every sequence in D with respect to sequence s. Formally:*

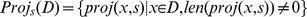
(1)


**Definition 7.** *Given a pair of sequences* 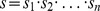 *and w, such that* 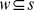 *, and* 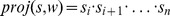 *, the* **pseudo-projection** *of s with respect to w, denoted as* 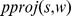 *is defined as follows:*

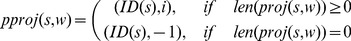
(2)
*where* 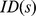 *gives a unique identifier for the sequence s. If the second entry of a pseudo-projection is* 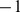, *the pseudo-projection will be called* **empty pseudo-projection**.

Roughly speaking, a pseudo-projection of a sequence *s* with respect to a sequence *w* stores a pair of values: the identifier of *s* and the start position of the projection of *s* with respect to *w*.

**Definition 8.** *Similarly to definition 6, given a sequence database D and a sequence s, the* **pseudo-projection database** *of D with respect to s, denoted as* 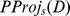 *is defined as the set of non-empty pseudo-projections of every sequence in D with respect to sequence s. Formally:*

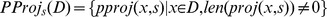
(3)


**Theorem 1.** Given a sequence database *D*, a pattern *p*, and a symbol 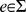, the support of 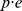, i.e., 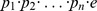, in *D* is equal to the support of *e* in the projection database of *D* with respect to *p*. Formally:
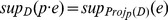
(4)


The formal proof for this theorem is similar to the one provided in [25] for a similar theorem. The proof is as follows:

*Proof.* (≥) Let *s* be a sequence in *D* that supports 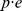. Then, *s* can be represented as 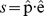, such that 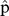 is the *p*-prefix of *s*, where 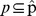 and 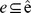. Thus, 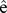 is the projection of *s* with respect to *p*, then 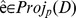. Also, as 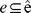 and 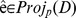, then for every sequence in *D* that supports 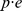, there will be a sequence in 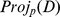 that supports *e*. This means that the support of *e* in 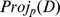 is at least equal to the support of 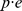 in D. Therefore, 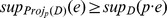.

(≤) Let 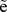 be a sequence in 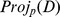 that supports *e*, that is, 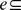 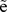. As 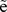 is the projection of a sequence *s*′ in *D* with respect to *p*, then 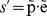, where 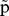 is the p-prefix of *s*′. Since 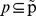 and 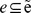, then 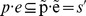. In this way, for every sequence in 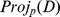 that supports *e*, there will be a sequence in *D* that supports 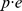. This means that the support of 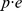 in *D* is at least equal to the support of *e* in 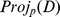. Therefore, 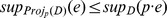.□

**Figure 1:**
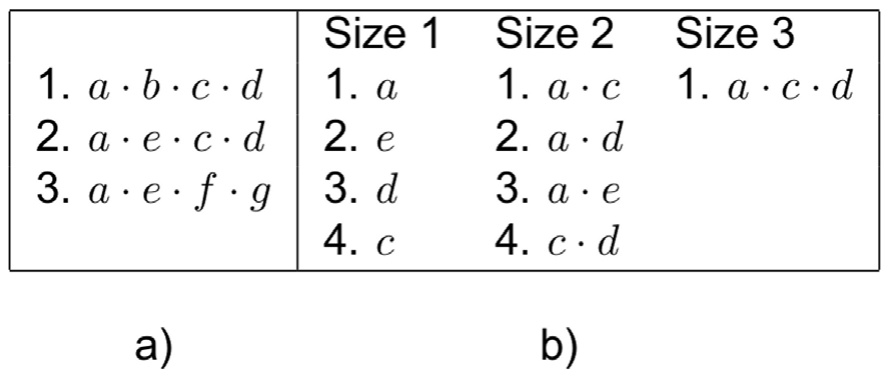
Example of a sequence database and its frequent sequences. Sequence database containing 3 sequences (a) and its correspondent frequent sequences (b), for a frequency threshold of 2.

Having defined the previous concepts, the **sequential pattern mining problem** can be defined as the task of finding all the frequent sequences in a given sequence database *D* with respect to a given threshold *ξ*. For instance, Figure 1 shows a database formed by 3 sequences, and its correspondent sequential patterns, for an absolute threshold (also called frequency threshold) of 2.

## Methods

**Figure 2:**
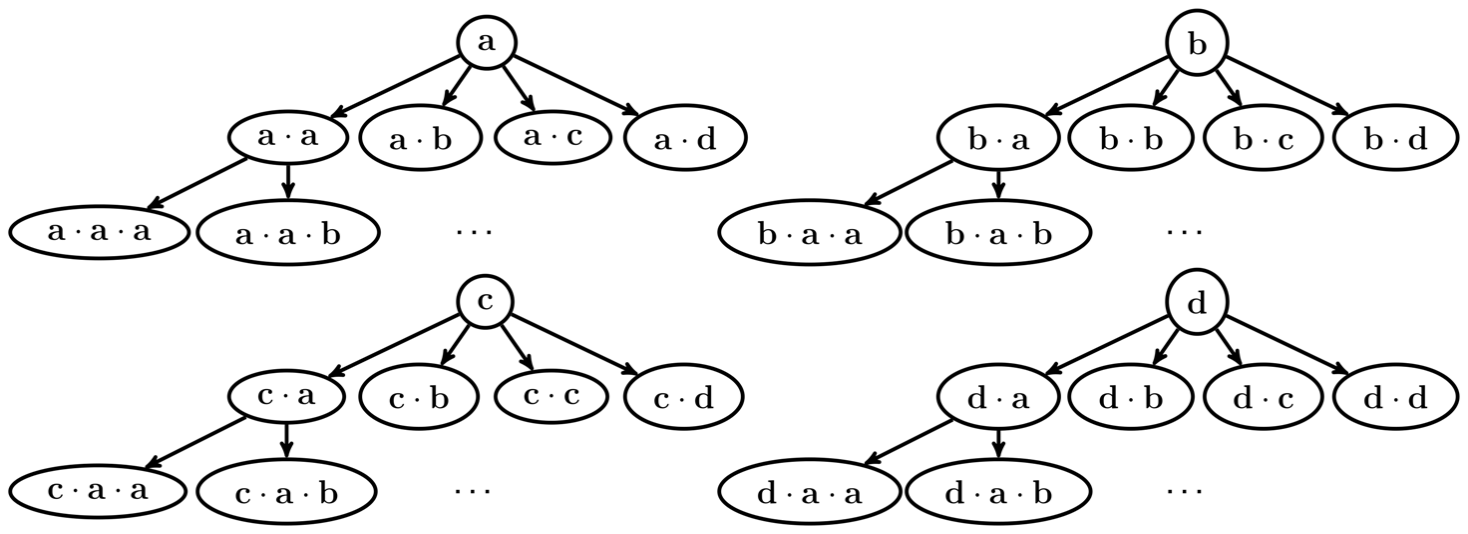
Example of search space. An example of the search space for a sequence database with 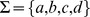.

The main idea behind the proposed algorithm is to build frequent sequences by growing an already known frequent sequence *p* with a frequent symbol *w* found in the database projection with respect to *p*, creating the sequence 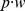, which Theorem 1 guarantees that it is a frequent sequence. This process repeats recursively, until there are no more frequent symbols to append to the current frequent sequence. The process then rolls back to the previous frequent sequence found and continues growing it with another frequent symbol. The search space, therefore, is represented as a series of trees, one for each frequent sequence of length 1. Each node of a tree represents a frequent sequence, and they are located in the tree in a way that a sequence node will be the same as its parent node, but with a symbol appended at the end of the sequence. Figure 2 shows an example of the search space of a small sequence database with four different symbols.

With each frequent sequence discovered, a new node is built and liked to its parent node. Each node contains a frequent sequence, and pointers to its parent node and its descendants, to maintain the structure of the current tree. Without loss of generality, the symbols in the implementation have been represented as positive integers. A sequence can be represented, then, as an array of integers.

Properly linking every frequent sequence found by the algorithm would result in a subtree of one of the search space trees. However, storing the whole tree in main memory during the mining process is not needed, only the branch where the sequence that is being grown belongs. This is because it is only required to access the ancestors of the current sequence, when the algorithm rollsback to a previous sequence and tries to grow it with another frequent symbol. This results in a significant memory saving, specially when dealing with a sequence database with a large set of different symbols (Σ).

Although the NLDFT algorithm does not generate candidate patterns, but grows frequent sequences from already found frequent sequences instead, the frequency of the found sequences still has to be ensured. To guarantee that, the proposed algorithm only uses symbols that meet the minimum support to build frequent sequences. Each time the algorithm search in a pseudo-projection database for symbols to build new frequent sequences, it only retrieves the symbols that meet the frequency threshold. In this way, when the algorithm uses those retrieved symbols to grow the current frequent sequence, it is assured that the new sequence is frequent as well.

The NLDFT algorithm is divided in three main steps. The first step comprises preliminary operations, which prepare the input data for the main mining process. The Algorithm 1 in Table 1 shows these operations. First of all, the algorithm reads the original database and finds the frequent symbols; these symbols correspond to the set of the frequent sequences of length 1. Next, the database is read again, and the symbols that are not on the set of frequent symbols are removed. Next, for each frequent symbols found, the pseudo-projection database with respect to that symbol is built. This is shown in Table 2. Next, a call to the mining process is made, having as input the symbol and the pseudo-projection database. The mining process then will recursively find the frequent sequences that start with the corresponding symbol. The union of the frequent sequence sets found with each iteration will constitute the set of the frequent sequences of input database.

**Table 1:** Algorithm 1.

**input**: *D*, a sequence database
*η*, a minimum support.
**output**: *F*, a set containing all the frequent sequences in D.
1 Initialization
//STEP 1: Find frequent symbols and filter input database.
2 FW ← GetFrequentSymbols (D, *η*)
3 MinFrequency ← D.size * *η*
4 MainDB ← FilterDatabase (D, FW)
5 MainProj ← GetPseudoProjection (MainDB, *−1*)
6 F ← FW
7 **foreach** *symbol a in* FW **do**
8 | Proj ← GetPseudoProjection (MainProj, *a*)
9 | F ← F ∪ Mine (Proj, *a*, *η*)
10 **end**
11 Return F

NLDFT's main function.

**Table 2:** Algorithm 2.

**input**: *D*, a pseudo-projection database
*a*, a symbol
**output**: ProjDB, the pseudo-projection database of *D* with respect to *a*
12 GetPseudoProjection (D, *a*)
13 {
14 ProjDB ← Ø
15 **foreach** *sequence d in* D **do**
16 | for *i* ← *d.Start * ***to*** D.*Size - 1* **do**
17 | | **if** *MainDB[d.ID][i] = = a* **then**
18 | | | Proj ← new ProjSequence Proj.ID = *d*.ID if *a = = −1* **then**
19 | | | | Proj.Start ← 0
20 | | | **else**
21 | | | | Proj.Start ← *i* + 1
22 | | | **end**
23 | | | ProjDB.add(Proj) break
24 | | **end**
25 | **end**
26 | delete Proj
27 **end**
28 return ProjDB
29 }

NLDFT's GetPseudoProjection function. This function creates a pseudo-projection database from an input pseudo-projection database and a symbol.

The second step of the algorithm occurs inside the main mining process, which receives as input data a pseudo-projection database, a sequence that will be grown to discover more frequent sequences and the minimum support. This step is shown in Table 3. The first operation made in the main mining process is finding the frequent symbols inside the input pseudo-projection database. This is done inside the function *GetFrequentSymbols*, which is shown in detail in Table 4. There, each sequence of the pseudo-projection database is read, and the occurrences of the symbols read in each sequence is counted. Once the function has read all the sequences, those symbols that met the minimum frequency are returned as output.

**Table 3:** Algorithm 3.

**input**: *D*, a pseudo-projection database
*p*, the growing sequence
*η*, the minimum support
**output**: *F*, a set containing all the frequent sequences in D.
30 Mine (D, *p*, *η*)
31 {
//STEP 2: Find frequent symbols in the given projection database.
32 FW ← GetFrequentSymbols (D, *η*)
//STEP 3: Form new frequent sequences by combining the sequence *p* with every symbol obtained in the previous step.
33 **foreach** *symbol a in* FW **do**
34 | F ← F ∪{*p* · *a*}
35 | Proj ← GetPseudoProjection (D, *a*)
36 | Mine (Proj, {*p* · *a*}, *η*)
37 **end**
38 Return F
39 }

NLDFT's Mine function. This function recursively grows the input sequence *p*.

**Table 4:** Algorithm 4.

**input**: D, a pseudo-projection database
*η*, the minimum support
**output**: FWArray, the set of frequent symbols in *D* with respect to *η*
40 GetFrequentSymbols (D, *η*)
41 {
42 **foreach** *sequence d in* D **do**
43 | Duplicates.clear()
44 | **for** *i* ← *sequence.begin* **to** *sequence.end* **do**
45 | | **if** Duplicates.exists (MainDB *[d.I D][i]*)* = = true* **then**
46 | | | Duplicates.add(MainDB [*d.ID*][i])
47 | | | **if** FWArray.exists (MainDB *[d.ID][i]*)* = = true* **then**
48 | | | | FWArray.count ++
49 | | | **else**
50 | | | | FWArray.add(MainDB [*d.ID*][i])
51 | | | | FWArray (w).count ← 1
52 | | | **end**
53 | | **end**
54 | **end**
55 **end**
56 **for** *wElem* ← FWArray.*begin* **to** FWArray.*end* **do**
57 | **if** *wElem.count* < *MinFrequency* **then**
58 | | wElem.delete
59 | **end**
60 **end**
61 return FWArray
62 }

NLDFT's GetFrequentSymbols function. This function finds the frequent symbols in the input pseudo-projection database.

The third and final step of the proposed algorithm also occurs inside the function *Mine*, and comprises building new frequent sequences out of the input frequent sequence and the frequent symbols found in step 2. After returning from the function *GetFrequentSymbols*, and for each of the symbols returned from it, a new frequent sequence is formed, by appending that symbol to the end of the input frequent sequence. Finally, a projection of the input database is generated and used as the input of a new recursive call to the *Mine* function, along with the new frequent sequence built, and the minimum support. The process continues recursively, until no frequent symbols are found in the current input database.

### Example

**Figure 3:**
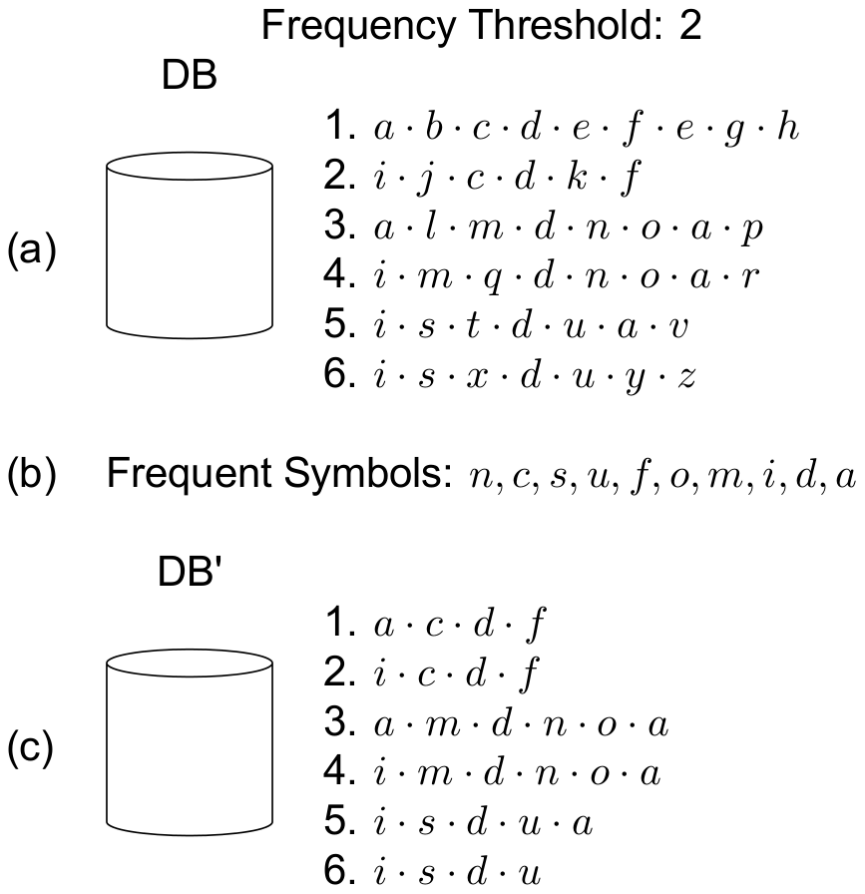
Example of the NLDFT algorithm's functionality (1 of 7). The figure shows a sequence database (a), the set of frequent symbols obtained from it (b) and the filtered database (c).

Next, an illustrative example about the proposed algorithm's functionality is shown. Figure 3 shows a sequence database composed by six sequences, which is going to be processed by the proposed algorithm with a frequency threshold of 2. The first step in the algorithm is to read the database and find the frequent symbols, which are also the set of frequent sequences of length 1. Next, the non frequent symbols are removed. The resultant database appears in the lower part of the figure.

**Figure 4:**
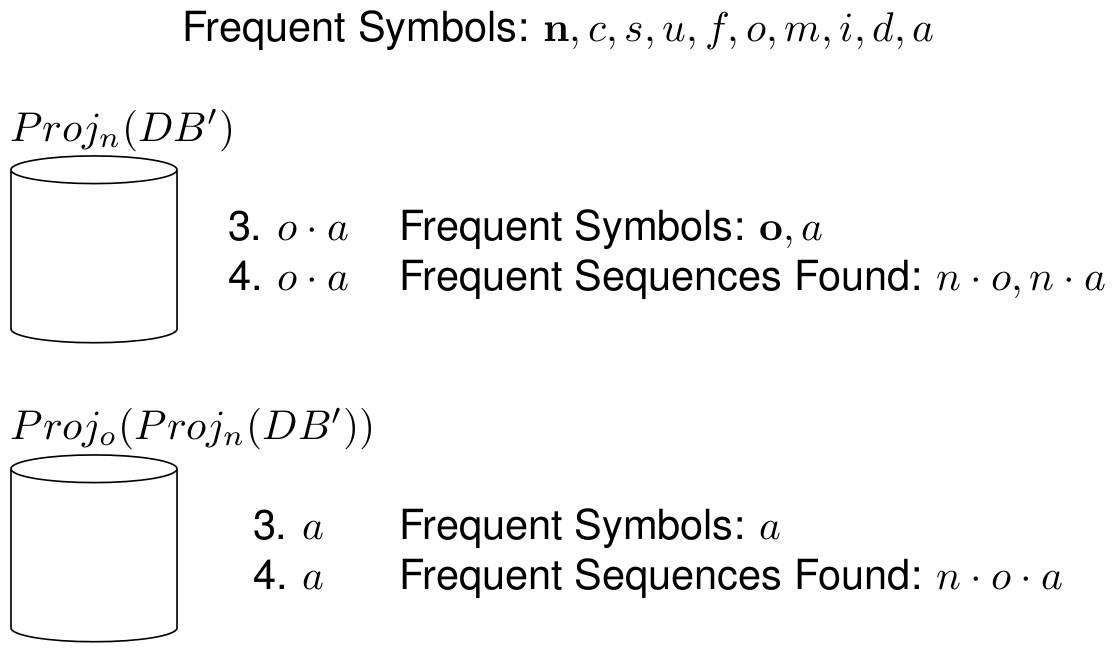
Example of the NLDFT algorithm's functionality (2 of 7). The figure shows two recursive calls to the main mining process, where 3 frequent sequences are found.

Next, one of the frequent sequences found is selected to be grown. This is shown in Figure 4. In this case, the chosen symbol is *n* and the pseudo-projection database with respect to that sequence is built. Next, the pseudo-projection database is searched for frequent symbols. From that search, two frequent symbols are found: *o* and *a*, which are used to build the frequent sequences 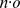 and 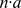. Next, one of the frequent symbols is chosen, in this case the symbol *o*, and a pseudo-projection database is built with respect to that symbol and the current database. Finally, this new pseudo-projection database is used, along with the symbol *o* and the sequence 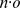 as the input for the next call of the mining process and the recursion continues; this part of the process corresponds to the Step 3 of NLDFT. From the input database of the new recursive call, the only frequent symbol found is *a*, which is used to build the frequent sequence 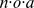.

**Figure 5:**
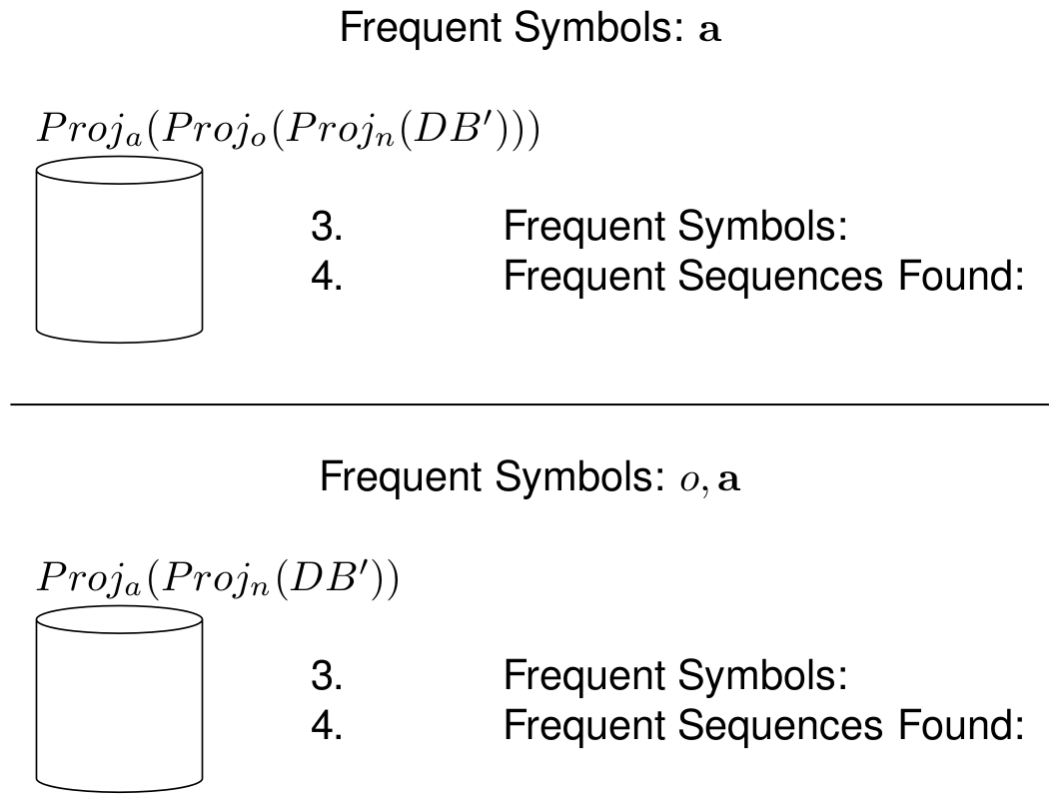
Example of the NLDFT algorithm's functionality (3 of 7). The upper part shows the next recursive call with respect to the Figure 4, where the input database does not contain any symbol. The algorithm goes back to the previous frequent sequence and makes another recursive call with a different frequent symbol (lower part of the figure), which does not contribute with any frequent sequence.

**Figure 6:**
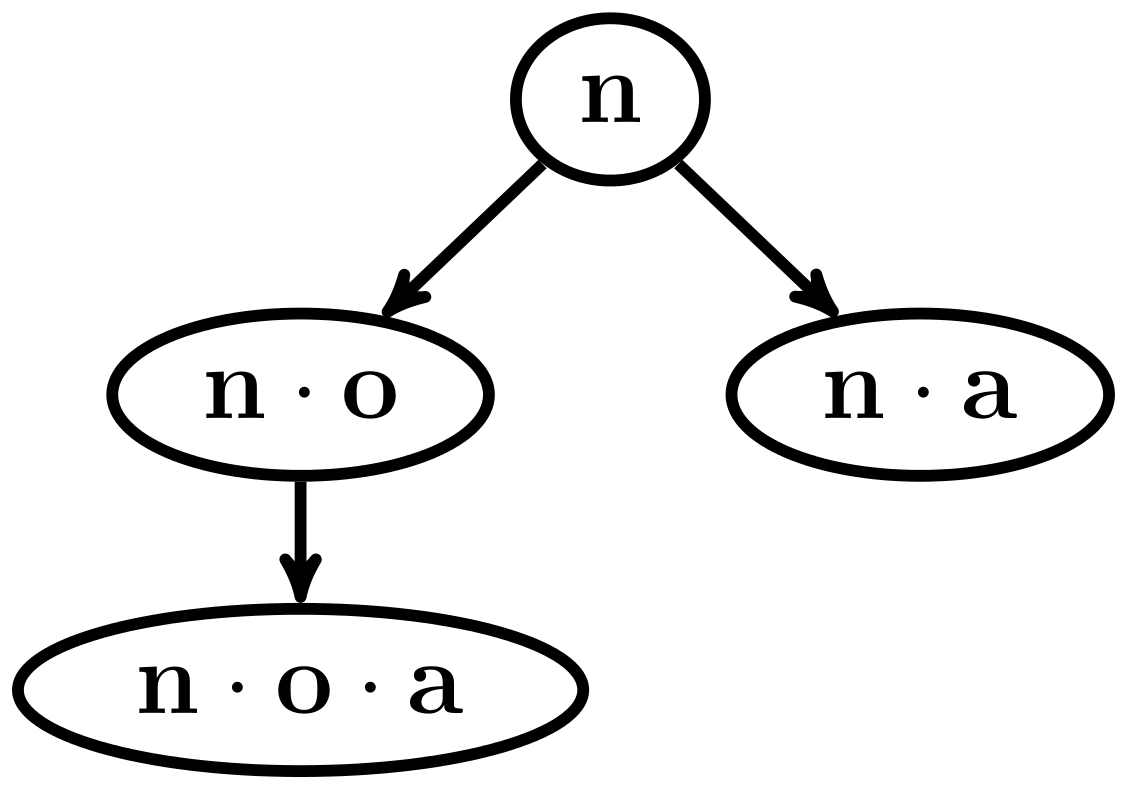
Example of the NLDFT algorithm's functionality (4 of 7). Frequent sequences found by growing the frequent sequence *n*.

**Figure 7:**
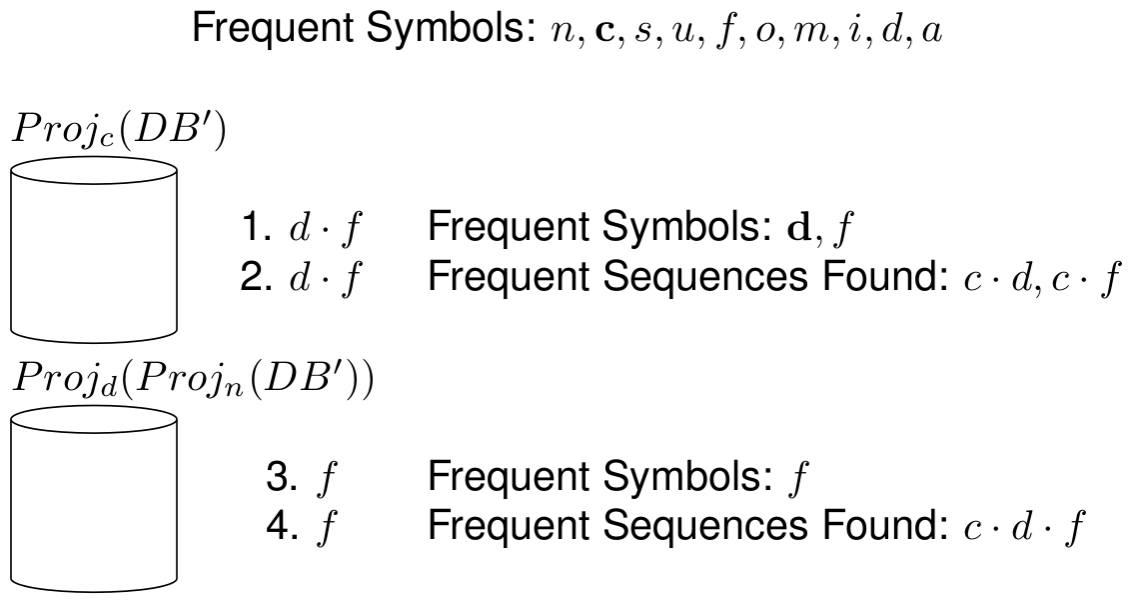
Example of the NLDFT algorithm's functionality (5 of 7). Recursive calls of the Mine function to grow the frequent sequence *c*.

**Figure 8:**
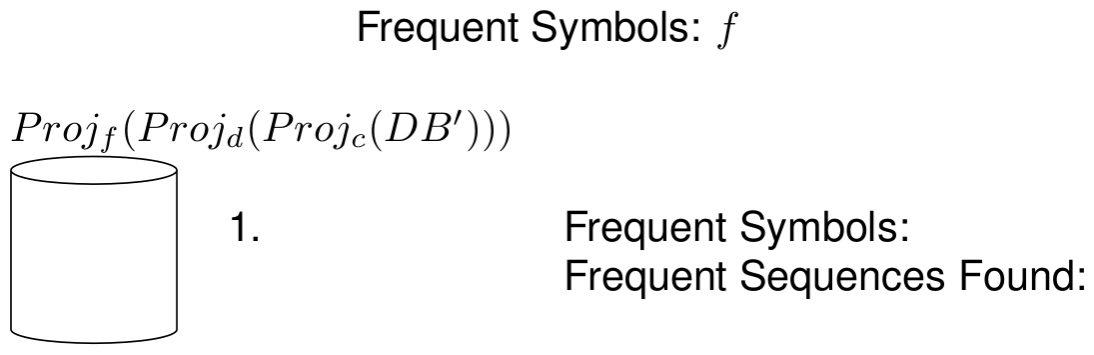
Example of the NLDFT algorithm's functionality (6 of 7). Last recursive call to grow the frequent sequence *c*. The projection database does not contain any symbol, so the recursion stops in this direction.

**Figure 9:**
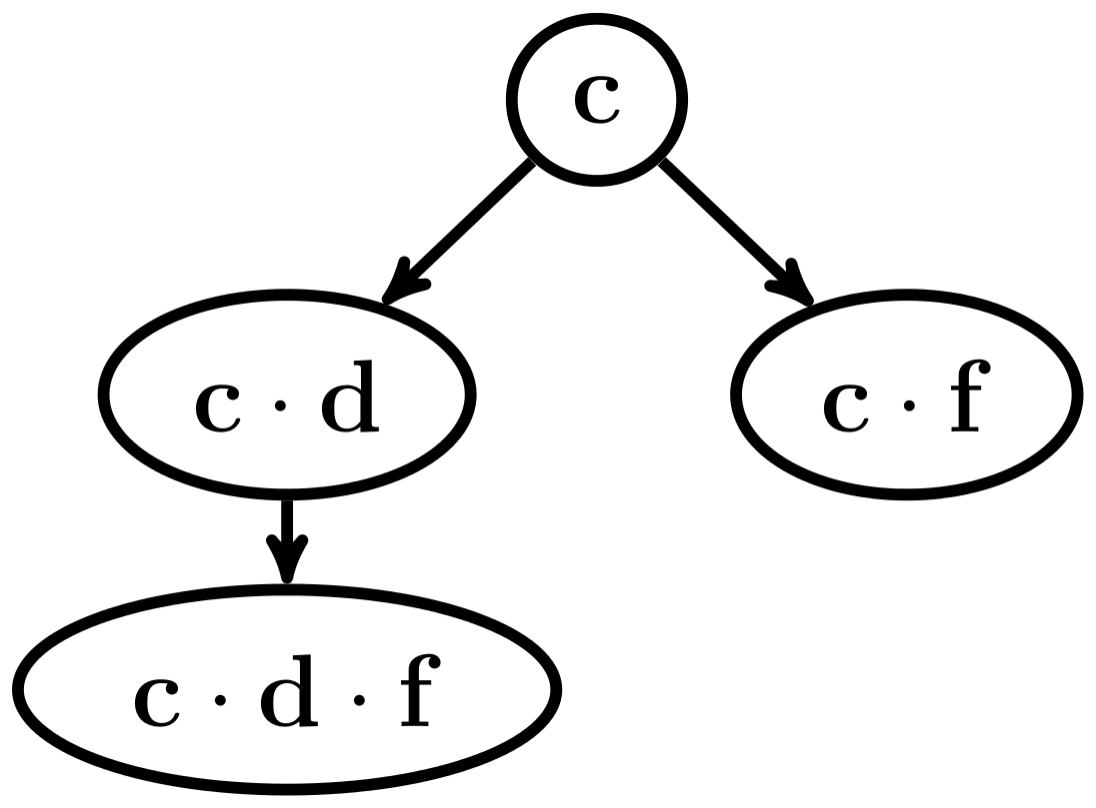
Example of the NLDFT algorithm's functionality (7 of 7). Frequent sequences found by growing the frequent sequence *c*.

In the next recursive call, there are no more frequent sequences to be found, as the input database does not contain any symbol. This is shown in the upper part of Figure 5. The algorithm then has to go back to a previous frequent sequence which still has unprocessed frequent symbols in its associated frequent symbol set, in this case the sequence *n*, and now choses the symbol *a* to use in a subsequent recursive call. Also, in this recursive call there are no frequent sequences to be found, because the input database does not contain any symbols. This is shown in the lower part of Figure 5. Because there are no more frequent symbols to grow the sequence *n*, all the frequent sequences that start with that symbol have now been found. These frequent sequences and their ancestor/descendant relations are shown as a tree structure in Figure 6. Next, the frequent sequence *c* is chosen and the same process as the one with the frequent sequence *n* is followed, which is shown in Figure 7. The pseudo-projection database is then built with respect to the frequent sequence *c* and frequent symbols are searched for in that database. The frequent symbols found are *d* and *f*, which are then used, along with the frequent sequence *c* to build the frequent sequences 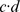 and 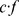. Next, the symbol *d* is chosen from the new frequent symbol set and another pseudo-projection database is generated. In this database, *f* is the only frequent symbol found, so the sequence 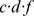 is the only frequent sequence built. Now, the just discovered symbol *f* is chosen and another pseudo-projection database is generated, which does not have any symbols, so the recursion stops there, and the process continues with the symbol *f* associated with the frequent sequence *c*, until all the frequent sequences that start with the symbol *c* are found. This is shown in Figure 8. These frequent sequences and their ancestor/descendant relations are shown in Figure 9. The next step is to chose another frequent sequence of length 1 and repeat the process. The algorithm will continue until all of the frequent sequences of length 1 are processed. Then, the complete set *P* of frequent sequences is: 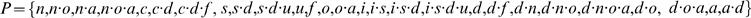.

The proof of the correcntess of the proposed algorithm is given in the next section.

### Correctness Proof

In this section, a proof is provided to demonstrate that the sequences found by the NLDFT algorithm in any given database and according to a minimum support form the complete set of frequent sequences. The correctness proof shows this in two parts: first proving that every sequence that meets the frequency threshold is found by the proposed algorithm; and second, that the proposed algorithm only discovers sequences that meet the given frequency threshold.

Let

- *D* be the input database.
- *η* be the frequency threshold.
- *P_alg_* be the set of frequent sequences discovered by the algorithm.
- *P* be the set of sequences that meet the frequency threshold.

The correctness of the proposed algorithm can be demonstrated by providing the following two statements:

- Statement 1: If *s* is a sequence that meets the frequency threshold, then *s* is discovered by the NLDFT algorithm, i.e., if 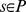 then 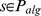.
- Statement 2: if *s* is a sequence discovered by the NLDFT algorithm, then *s* meets the frequency threshold, i.e., if 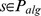 then 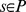.

#### Proof of Statement 1

It will be proven that if 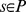 then 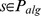, by mathematical induction over the length of *s*, 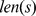, for all the natural numbers, 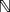.

*Proof*.

**Basis**: Show that the statement holds for 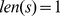. The algorithm scans the input database and finds all the symbols whose frequency is equal or greater than the frequency threshold as a first step. These symbols correspond to the frequent sequences of length 1. Therefore, if *s* meets the frequency threshold, it is found by NLDFT, which shows that the statement 1 holds for the basis step.

**Inductive step**: Show that if the statement holds for a sequence *s*′ of length *k*, then it also holds for a sequence *s* of length *k*+1 where 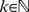.

Starting from the sequence *s*:
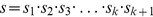
(5)


This could be rewritten as:
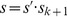
(6)


Since 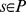, then 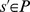 and 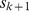 is frequent. Also, *s*′ is found by the NLDFT algorithm, by the induction hypothesis. Moreover, in the Step 2 of NLDFT (shown in Algorithm 3, Table 3), the algorithm generates a list of symbols that meet the frequency threshold for the pseudo-projection database 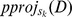. Also, by Theorem 1, it is known that 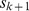 also meets the frequency threshold for the pseudo-projection database 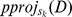. Therefore, 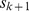 is included in the list of symbols generated in step 2, and then it will be appended at the end of *s*′ in step 3, forming the frequent sequence *s*. Thus, it has been shown that 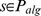.□

#### Proof of Statement 2

It will be proven that if 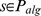 then 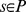, by mathematical induction over the length of *s*, 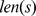, for all the natural numbers, 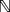.

*Proof*.

**Basis**: Show that the statement holds for 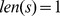.

As 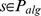 and 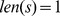, the NLDFT algorithm finds *s* in Step 1 (shown in Table 1), as it is in this step where all the frequent sequences of size 1 are discovered. Also, 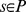, as the NLDFT algorithm only retrieves the symbols that meet the frequency threshold. Thus, it has been shown that the statement 2 holds for the basis step.

**Inductive step**: Show that if the statement holds for a sequence *s*′ of length *k*, then it also holds for a sequence *s* of length *k*+1 where 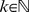


Starting from the sequence *s*:
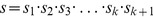
(7)


This could be rewritten as:
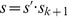
(8)


Since 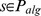, then 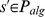 and 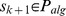. The NLDFT algorithm finds 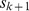 in the Step 2 (shown in Algorithm 3, Table 3), as part of a set of frequent symbols in 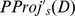, discovered with the function 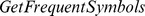. Therefore, 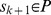. Next, in step 3, the symbol 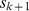 is appended at the end of the sequence *s*′, forming the sequence *s*. By the inductive hypothesis, 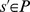. Also, by the Theorem 1, it is known that if 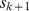 meets the frequency threshold in the pseudo-projection database 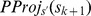, then the sequence 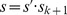 also meets the frequency threshold in *D*. Thus, *s* meets the minimum support. Therefore, it has been shown that 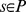.□

### Complexity Analysis

The running time of the sequential pattern mining problem depends not only in the dimensions of the input database and the minimum support, but in the form of the sequences as well, and it is unfeasible to consider an average case scenario, because that would depend on the domain of the input database. Therefore, a worst case scenario will be considered to analyze the NLDFT algorithm's performance.

**Figure 10:**
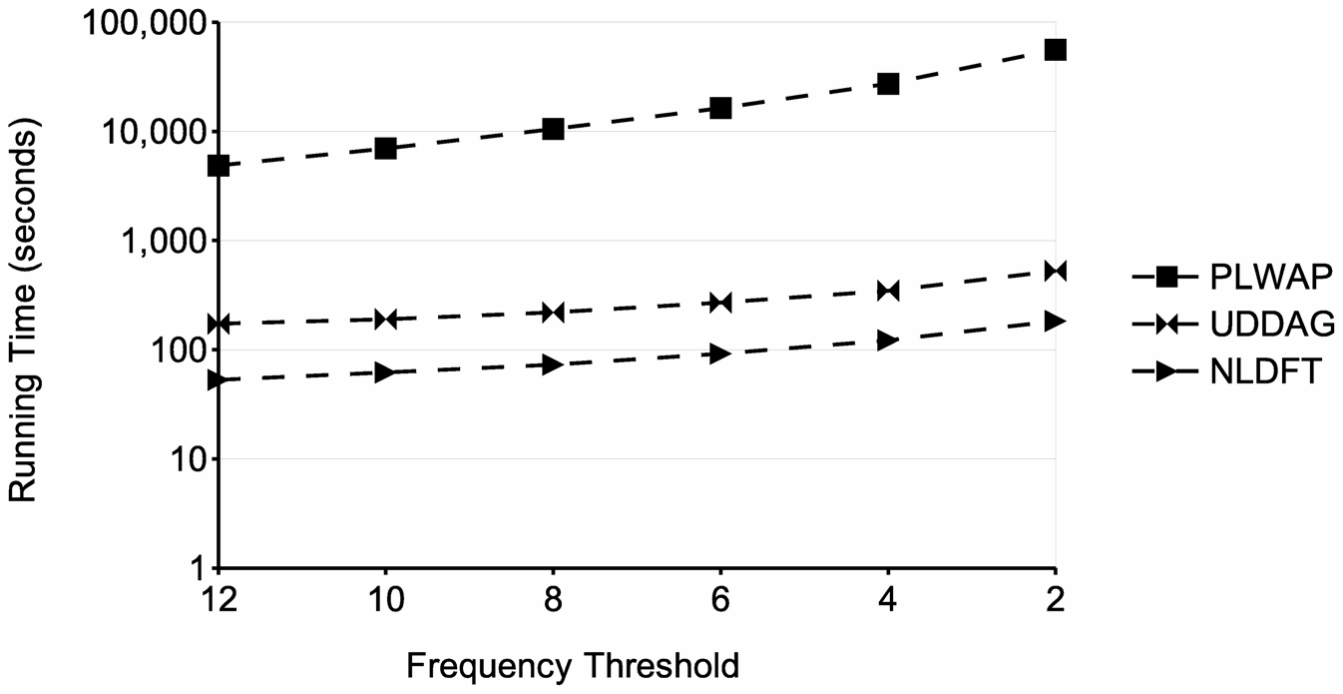
Comparison of runtime for different frequency thresholds. Processing 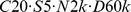.

**Figure 11:**
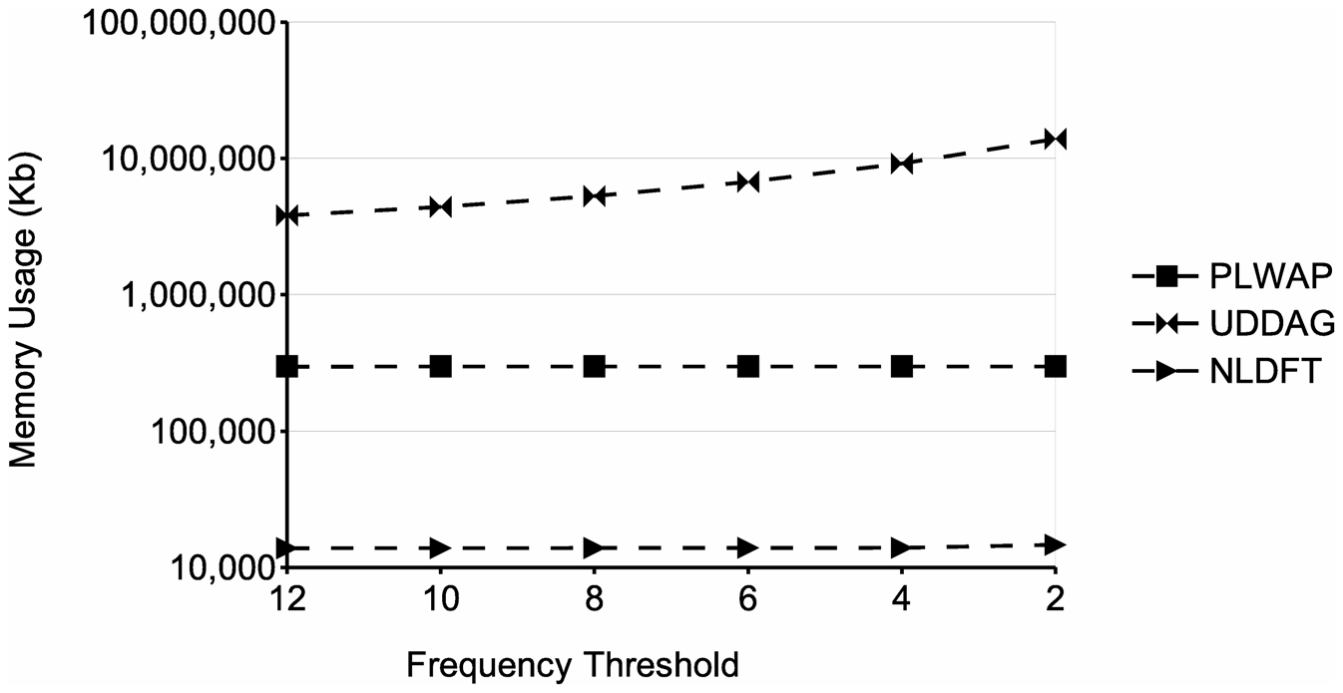
Comparison of runtime for different frequency thresholds in the study case. The figure shows the runtimes obtained when processing the study case database at frequency thresholds varying from 20 to 10 sequences.

**Figure 12:**
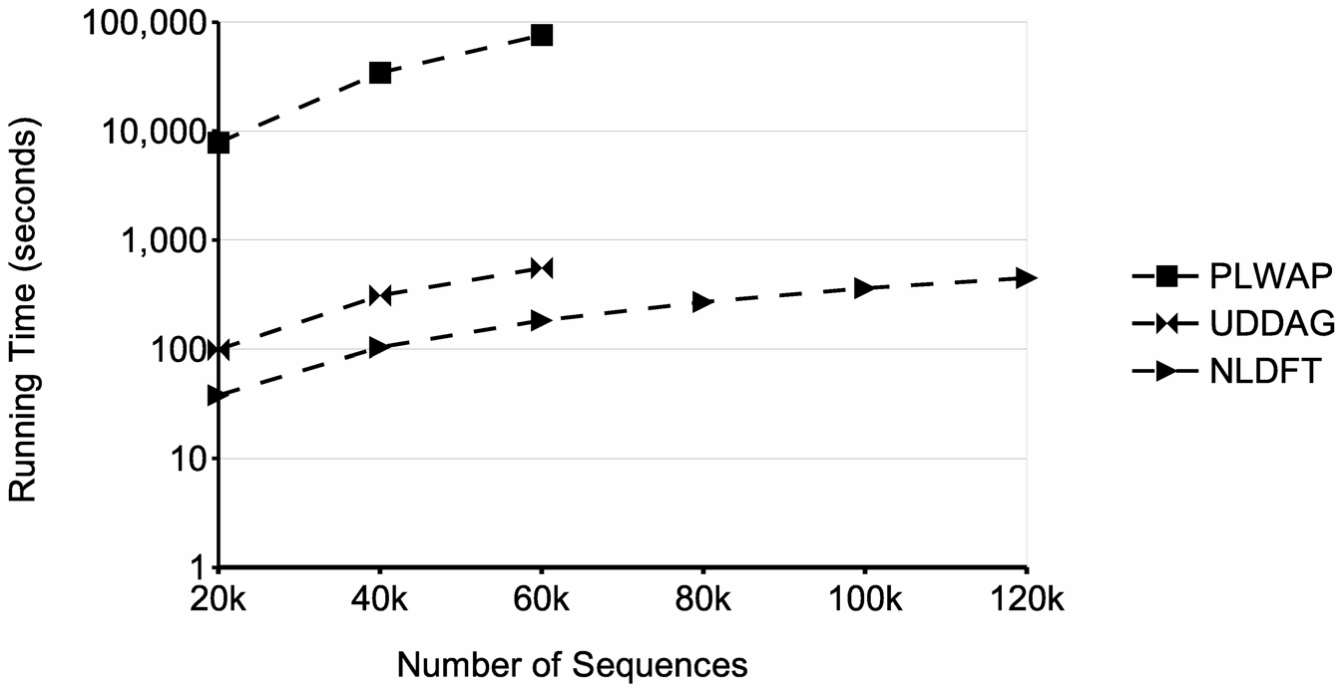
Example of an input sequence database in a worst case scenario. The figure shows a sequence database of size 4×4 (a)), formed by two pairs of sequences with different symbols, unique to each pair. The number of patterns of each pair is equal to the size of the power set (minus the empty set) of the set formed by the symbols in each pair (b)). The set of frequent sequences *P* in the database is the union of the sets of frequent sequences in each pair, 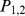 and 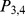.

Since lowering the minimum support yields a larger set of frequent sequences and increases the running time of the mining process, (see Figure 10 and Figure 11), the chosen frequency threshold in a worst case scenario will be the minimum, i.e., a threshold of 2 sequences. Considering this, a worst case scenario for an input database of size *mn* will have 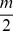 pairs of sequences, each of size *n*, and each pair of sequences will be composed by different symbols, unique to that pair. This would yield for each pair of sequences a set of frequent sequences with size equal to the size of the power set of its symbols (minus the empty set), which is the maximum number of frequent sequences that can be discovered in each pair of sequences. The union of the sets of frequent sequences of each pair of sequences will form the final set of frequent sequences. Thus, for a database of size *mn* and a frequency threshold of 2, the size of the set of frequent sequences is given by 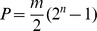. An example of an input database for a worst case scenario is shown in Figure 12.

The two main operations of the NLDFT algorithm are finding the frequent symbols in a given pseudo-projection database and building pseudo-projection databases. Since finding the frequent symbols entails checking every symbol in a given pseudo-projection database, at most *mn* symbols have to be checked. Since checking a symbol takes constant time, the time needed to complete the entire operation is O(*mn*). Similarly, to build a pseudo-projection database entails checking at most every symbol in the previous pseudo-projection database, and to process a symbol takes a constant number of operations, thus the time needed to build a pseudo projection is also O(*mn*). Furthermore, these two main operations have to be performed everytime a recursive call is made. Since a recursive call is made for every frequent sequence discovered, in a worst case scenario 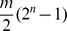 recursive calls are made. Therefore, in a worst case scenario, the running time of the NLDFT algorithm is 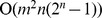.

It is difficult to theoretically compare the performance of the NLDFT algorithm with the ones of the UDDAG and PLWAP algorithms. The authors of the UDDAG algorithm do not give a final running time bound of the algorithm, but only an upper bound on the number of projections made and a description of the operations made for the other parts of the algorithm. Although it appears that the UDDAG builds less database projections than the NLDFT algorithms, since it grows a pattern in both directions at each recursion level instead of one, it has to go through a candidate generation operation at each recursion level as well, which severely increases the running time. On the other hand, the authors of the PLWAP algorithm state that the time to build the PLWAP-tree is 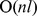, where *n* is the number of frequent symbols and *l* is the length of the longest frequent sequence in the given database, and the time to perform the mine process is 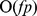, where *f* is the number of frequent symbols and *p* is the number of frequent sequences. However, is not clear if those bounds are of an average or worst case scenario. In a worst case scenario, the running time of the PLWAP's mining process is 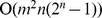, which is the same as the NLDFT algorithm's time complexity. This made necessary to perform running time experiments, in order to better understand the performance of the three algorithms and how they compare with each other.

**Figure 13:**
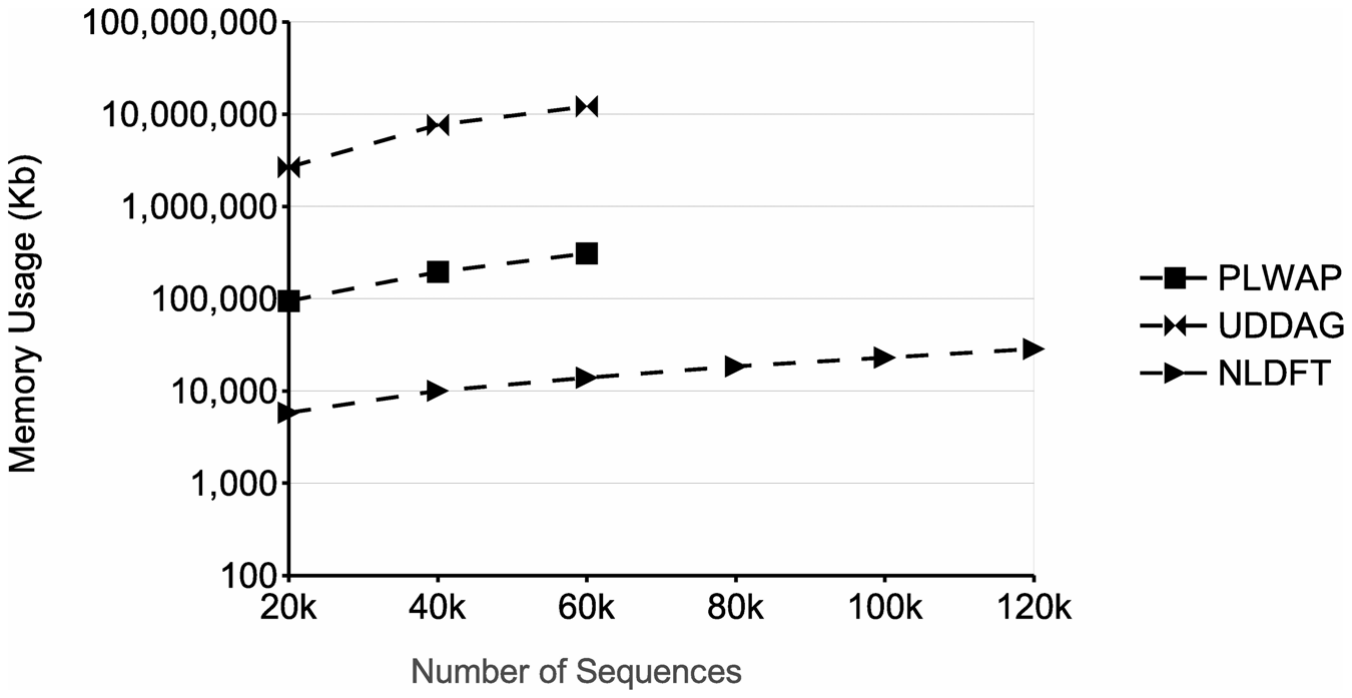
Comparison of memory usage for different number of sequences. The figure shows the memory usage obtained when processing databases with a number of sequences varying from 20k to 120k.

**Figure 14:**
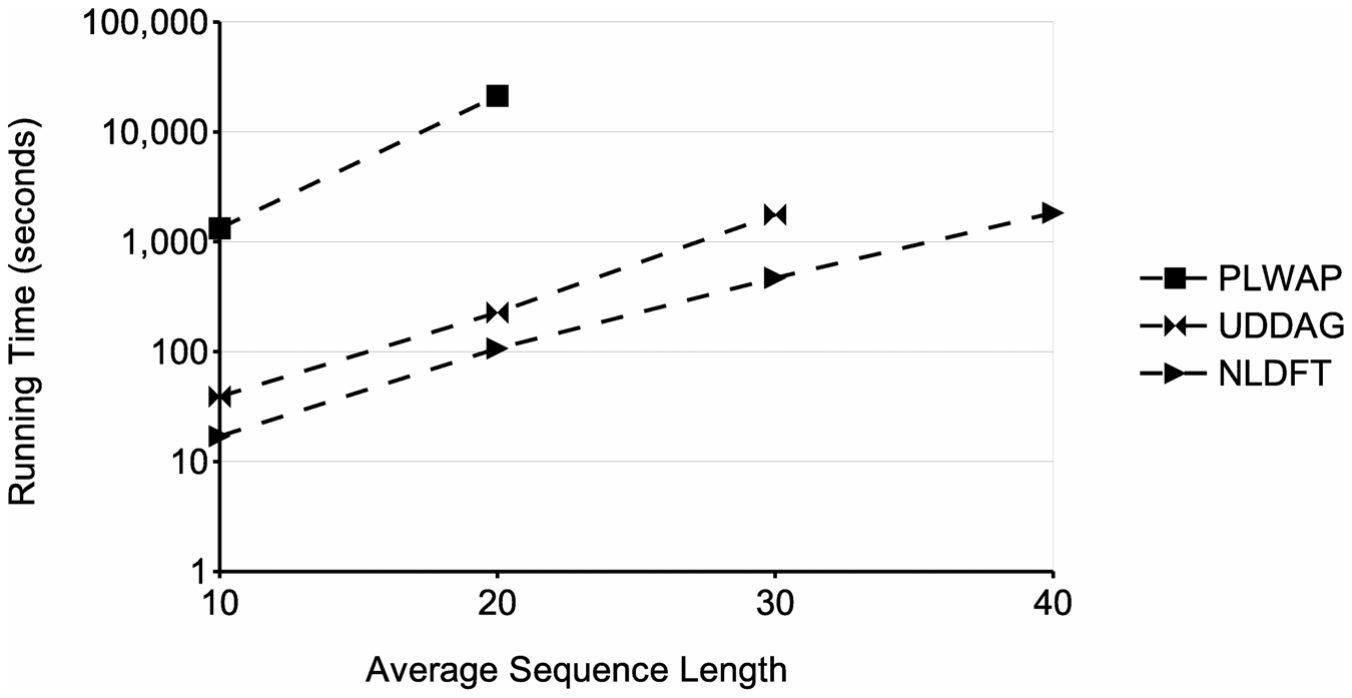
Comparison of memory usage for different average sequence lengths. The figure shows the memory usage of the tested algorithms when processing databases with an average sequence length varying from 10 to 40 symbols.

From the memory requirements' point of view, the NLDFT algorithm maintains in main memory the filtered input database (without nonfrequent symbols), the node linkage of the pattern being growth, along with the set of frequent symbols and the pseudo-projection database associated with each node. Moreover, each pseudo-projection database and each frequent symbols set are smaller than the ones of the previous node, and once the frequent sequence being processed cannot be grown, its associated frequent symbols set and pseudo-projection database are deleted, releasing the occupied memory. On the other hand, the UDDAG also uses pseudo-projections to grow frequent sequences, but as it grows sequences bidirectionally, it has to store an extra pointer for each sequence in the pseudo-projection database, to indicate no only the start position of the sequence in the input database, but the ending position as well. Also, at each recursion level, the UDDAG algorithm generates candidate sequences, which are then stored and tested against the minimum support. The number of candidates stored severely increases when dealing with large databases and/or low minimum supports, which has a very negative impact on the memory consumption of the entire algorithm (see for instance Figures 13 and 14). The PLWAP algorithm, on the other hand, does not rely on pseudo-projection databases and does not maintain the input database in main memory during the mining process. However, the PLWAP algorithm builds and maintains during the whole mining process an elaborated data structure. When dealing with large databases and/or low minimum supports, this data structure occupies a significant amount of memory. This is because the tree structure built has a branch for every sequence in the input database. Also, a queue is created for each frequent symbol, which contains all the occurrences of each frequent symbol in the tree structure.

## Results and Discussion

### Experimental Setup

A series of experiments were conducted to compare the proposed algorithm against representative approaches. The proposed algorithm was compared against the UDDAG [13] and PLWAP [2] algorithms. According to the authors of [13], the UDDAG algorithm is considerable faster than representative algorithms such as PrefixSpan, LapinSpan [26] and SPADE. Moreover, according to [18], PLWAP is the best model of pattern-growth tree projection sequential pattern mining techniques.

The PLWAP algorithm's code was obtained from the author's web page and the UDDAG algorithm was programmed. Both algorithms were coded in C++ and were compiled with g++. The proposed algorithm was also programmed in C++, and the experiments were performed in a computer with a core i7 processor of 3.40 GHz, 16 GiB of RAM and using GNU/Linux with the Ubuntu 11.10 distribution, as operating system. To perform the experiments, synthetic sequence databases were used, which were created with MBSDG (*Market-Basket Synthetic Data Generator*), an open source application available at [27]. The application is based on the IBM Quest application, which was used to generate test databases for several sequential patter mining approaches (e.g. [2], [13], [1], among others). The following parameters were used to generate the databases:

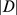
: Number of sequences in the database.

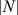
: Number of different symbols in the database.

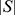
: Average length of maximal potentially frequent sequence.

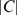
: Average sequence length.

For instance, a database labeled as 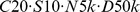 means that 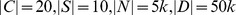, i.e. a database with an average sequence length of 20 symbols, an average of 10 symbols length for the maximal potentially frequent sequence, 5,000 different symbols and 50,000 sequences. The increase of any of this parameters when generating a database will cause an increase in the execution time when finding sequential patterns in such database. The parameters were chosen to be the same as those used in [2] for testing the PLWAP algorithm, in order to have similar experimental conditions. However, the minimum supports used in this paper are lower than those used in [2], as its computational resources were more limited than the ones used in this article. The database format consists of a single file, with a line of positive integer numbers per sequence. The numbers correspond to the symbols of the sequences and are separated by spaces. No additional information about the database should be provided to the algorithm.

**Figure 15:**
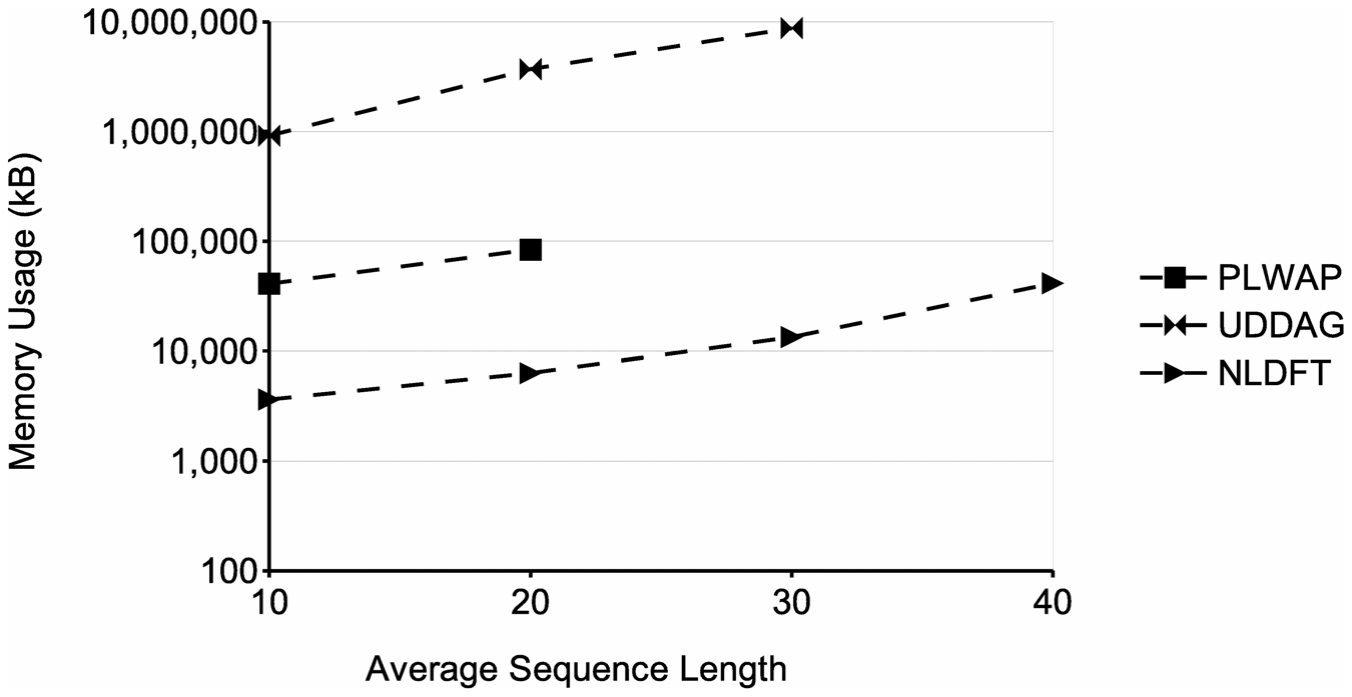
Comparison of memory usage for different frequency thresholds. Processing 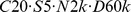.

Figure 10 shows a comparison of running time for different frequency thresholds, using a database with parameters 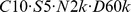. The graph shows that the processing time of the three algorithms increases as the minimum support decreases. This is because with lower supports, the number of patterns that meet the frequency threshold increases, which entails more processing. However, the proposed algorithm performs significantly better than the other two algorithms, even having a running time of two orders of magnitude lower than the PLWAP algorithm. Also, Figure 15 shows the memory usage of the three approaches for the same tests. In this experiment, the NLDFT algorithm also requires less memory than the other two algorithms, even being two orders of magnitude below the UDDAG's memory. Also, although the UDDAG algorithm had a better running time than the PLWAP algorithm, it is much more memory consuming and this consumption significantly increased when the frequency threshold was lowered.

**Figure 16:**
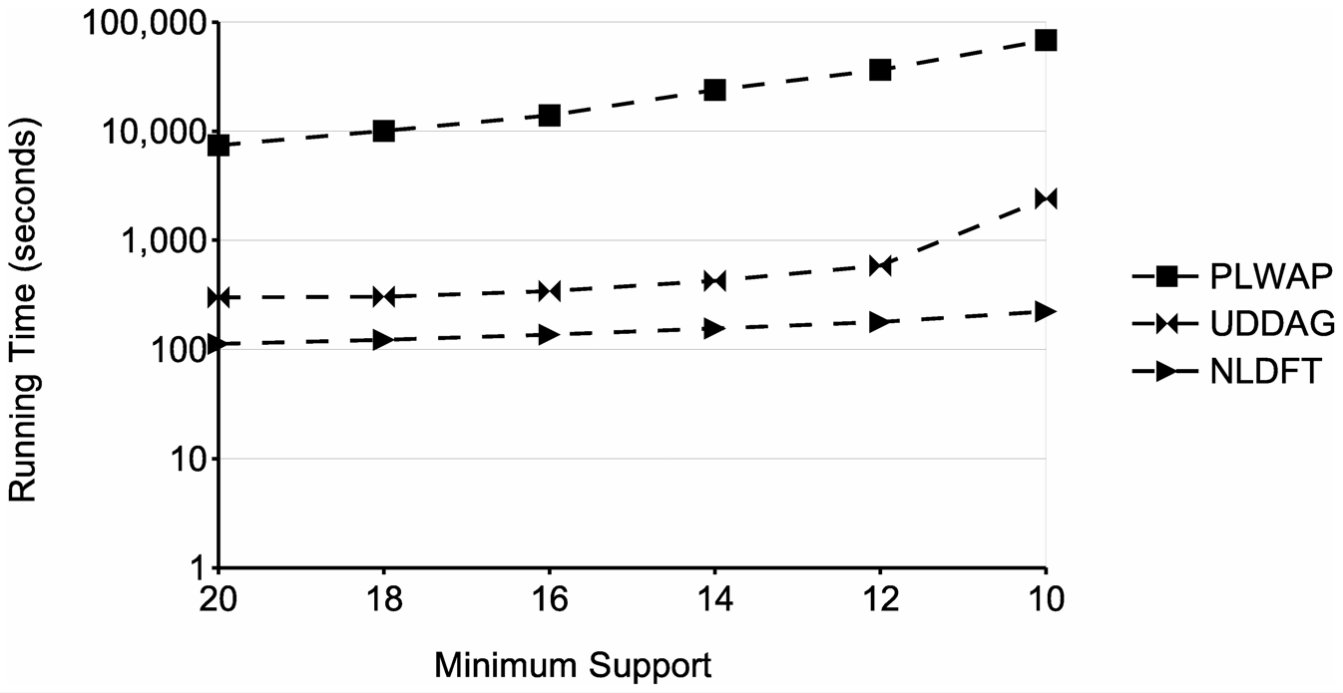
Comparison of runtime for different number of sequences. The figure shows the runtimes obtained when processing databases with a number of sequences varying from 20k to 120k.

Figure 16 shows a comparison of running time for processing databases with different number of sequences. These databases were generated using 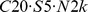 for the first three parameters and 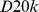, 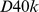, 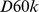, 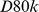, 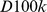 and 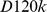 the last parameter, respectively. The frequency threshold was fixed at 2. The graph shows that as the number of sequences increases, the running time of the algorithms increases as well, as more patterns are likely to be discovered and more sequences have to be mined. When dealing with the database of 80 k sequences, the UDDAG algorithm ran out of memory. This is likely due the candidate generation phase that the UDDAG algorithm has to go through at each level of recursion, because it generates and stores a large amount of candidate sequences, and this amount gets larger when dealing with large databases and/or lower minimum supports. Moreover, the PLWAP algorithm did not finish in an acceptable time (less than 24 hours). The NLDFT algorithm, on the other hand, had a better running time than the other two algorithms and was capable of dealing with the largest database used in the experiment. Figure 13 shows the memory usage of the three approaches in this experiment. The memory consumption of the three approaches increased when dealing with larger datasets. In this experiment, the PLWAP algorithm also had a better memory consumption than the UDDAG algorithm, although the UDDAG had a better running time. The NLDFT algorithm also had a better memory consumption than those two algorithm, and always maintained the consumption below 100 MB.

**Figure 17:**
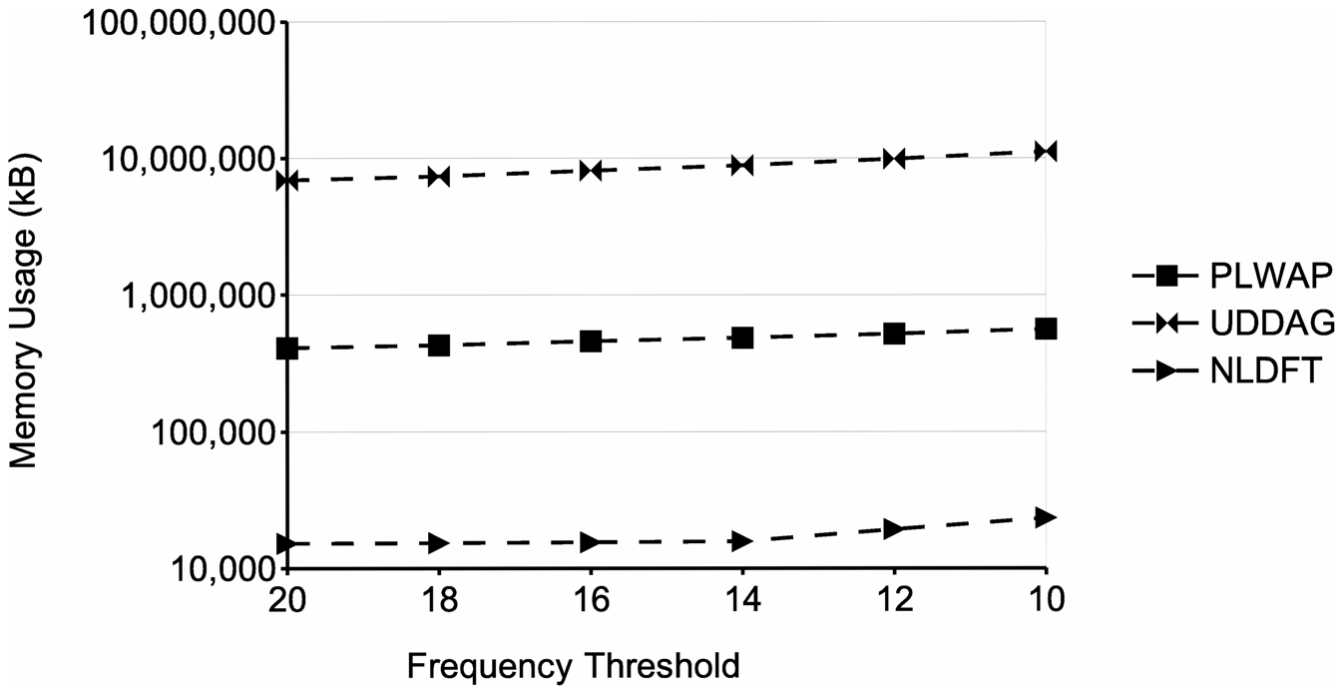
Comparison of runtime for different average sequence lenghts. The figure shows the runtimes obtained when processing databases with an average sequence length varying from 10 to 40 symbols.

Figure 17 shows the running times of the algorithms when processing databases with different average sequence lengths. Four databases were used, with parameters 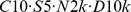, 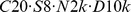, 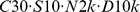 and 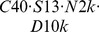 and a frequency threshold of 2. The graph shows that the running times of the three algorithms increase with the average sequence length. This is likely because larger sequences can contain more patterns than smaller ones, and the patterns could be larger as well, which entails more processing and storage of these patterns. In this experiment, the NLDFT algorithm performed better than the other two algorithms and was capable of dealing with the largest average sequence length tested. When dealing with the database with an average sequence length of 30, the PLWAP did not finished in an acceptable time (less than 24 hours). Also, the UDDAG algorithm ran out of memory when dealing with the dataset with an average sequence length of 40 symbols. As mentioned before, this is likely because the amount of candidate sequences the UDDAG generates and stores at each level of recursion greatly increases when dealing with larger databases. Figure 14 shows the memory usage of the three approaches for this experiment. The graph shows that the memory consumption increases when dealing with larger average sequence lengths, as more and larger patterns have to be stored. The NLDFT algorithm had a better consumption than the other two algorithms in this experiment as well, and maintained its consumption below 100 k kB in all the tests. Finally, Table 5 shows the number of patterns found by the three algorithms in each database used in the experiments.

**Table 5:** Number of patterns per database.

Database	Frequency Threshold	Patterns
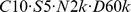	12	37,510
	10	55,941
	8	86,748
	6	142,563
	4	255,800
	2	700,242
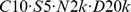	2	237,493
D40k	2	474,544
D60k	2	700,242
D80k	2	912,238
D100k	2	1,164,148
D120k	2	1,428,286
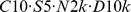	2	115,714
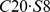	2	854,718
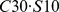	2	4,735,950
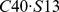	2	21,848,524

Number of patterns obtained with each tested database and frequency threshold.

### A study case: text documents as a sequence database

The earliest sequential pattern mining algorithms were applied to databases comprised by web logs, where each sequence was formed by a series of web pages visited by a user in a period of time, or customer transactions, where each sequence was formed by the items bought by one customer in a period of time. However, sequential pattern mining algorithms can be applied to any database comprised by sequences. In the case of text documents, as text keeps a natural sequential order given by its characters and words, its possible to apply a sequential pattern mining algorithm to a set of documents, where a sequential element can be a single character, a word or even a contiguous sequence of items (*n-gram*), and the sequential order is given by the occurrence of such element in the document. Finding frequent sequences of text is an interesting task because the found patterns could contain information about the author's style, fixed expressions, similitude between documents, among others. Moreover, text corpora poses a challenge for current sequential pattern mining algorithms, because these datasets are usually comprised of a large number of documents, which can be of a large size. The above was the motivation to peform a series of experiments using a text database, which results are presented in this section.

In this study case the behaviour of the proposed algorithm was tested when dealing with text documents and also aimed to find the terminology that represents the concepts of the documents' domain.

The dataset is composed of 57,653 text sequences with an average length of 20 symbols or words and a symbol set size of 28,547 symbols, that belong to a set of scientific articles of the oil extraction domain. To prepare the corpus, the stopwords were removed and a numerical representation was generated, where a unique integer was assigned to each different word.

**Figure 18:**
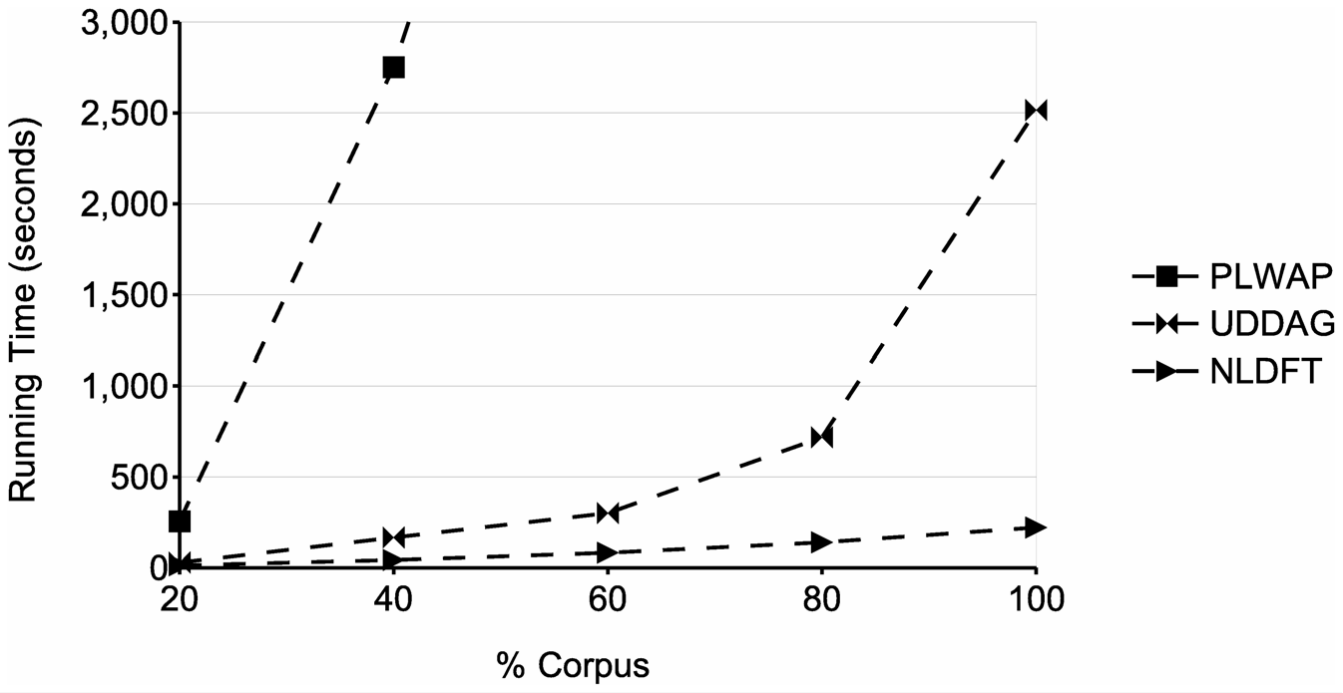
Comparison of memory usage for different frequency thresholds in the study case. The figure shows the memory usage of the tested algorithms when processing the study case database at frequency thresholds varying from 20 to 10 sequences.

Figure 11 shows a comparison between the PLWAP, UDDAG and the proposed algorithm at different minimum supports. The figure shows the running times obtained from tests done with minimum supports from 20 to 10; when tested with a lower support, UDDAG and NLDFT algorithms ran out of memory and PLWAP was stopped after a week of processing. However, the proposed algorithm performed significantly better than the other two methods, specially at lower minimum supports. Moreover, the running time of the NLDFT algorithm increased with less pronunciation than the other two algorithms, when decreasing the frequency threshold. Figure 18 shows the memory usage of the three algorithms in the previous experiment. The graph shows that the UDDAG algorithm consumed much more memory than the other two algorithms, even at the highest minimum support tested, in which it consumes almost 6 gigabytes of memory. Also, the proposed algorithm consumed significantly less memory than the PLWAP algorithm, going from 25 megabytes to 87 megabytes approximately, at the lowest support in the graph, compared with the PLWAP algorithm, which starts at 390 megabytes and goes up to 547 megabytes approximately. This is likely because the PLWAP algorithm builds a tree data structure and queues for each frequent symbol, representing the input database, which could consume a great amount of memory when the input database is large, and maintains it during the whole mining process.

**Figure 19:**
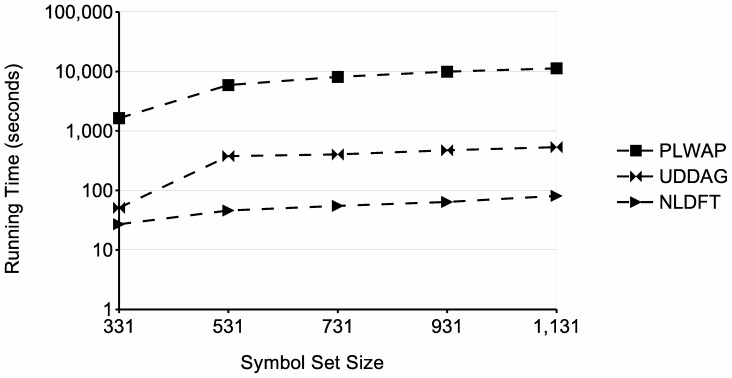
Comparison of runtime at different percentages of the corpus in the study case. The figure shows the runtimes of the tested algorithms, at different percentages of the study case database.

**Figure 20:**
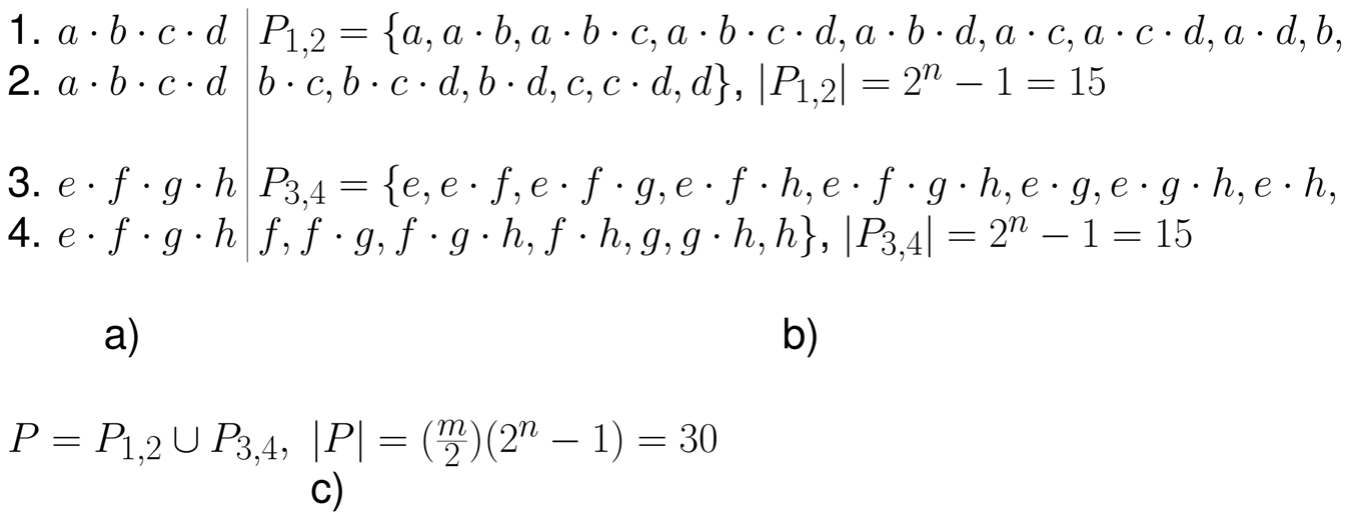
Comparison of runtime at different symbol set sizes in the study case. The figure shows the runtimes of the tested algorithms, at different sizes of the symbol set of the study case database.

Figures 19 and 20 show the running time during experiments done to test the scalability of the proposed algorithm. In the experiment of Figure 19, algorithms PLWAP, UDDAG and NLDFT's running time were tested using different percentages of the corpus, and a fixed frequency threshold of 10 sequences. It is important to notice that even at 40% of the corpus, the PLWAP algorithm tooks almost five times the running time of PLWAP and UDDAG algorithms, and tends to exponentially increase its running time as the number of sequences increases. UDDAG and NLDFT algorithms showed a similar performance until dealing with the 80% of the corpus, which is were the UDDAG algorithm takes almost two times the running time of NLDFT, and this difference increases when dealing with the 100% of the corpus. This is likely because when dealing with larger databases, the candidate-generation phase of the UDDAG algorithm generates a huge amount of candidate patterns, which testing consumes a significant amount of time.

Figure 20 shows the running time obtained when varying the size of the symbol set (Σ). This was done by removing the least frequent symbols, those that occurred in less than a hundred sequences, and only maintaining those symbols that occur in at least one hundred sequences. The resultant symbol set had a size of 1131 symbols. From this symbol set, several test were done, by removing symbols from the set by its frequency, in an ascending order. The frequency threshold was fixed to 10 sequences. The graph shows that, when dealing with a larger symbol set, the running time of the three algorithms increased. This is likely because, as more frequent symbols were added, more frequent patterns arised, which entailed more storage and processing. Moreover, there is a significant difference between the running times obtained in the test done with a symbol set of 331 symbols and the test done with a symbol set of 531. This is because the number of frequent sequences that were created when adding the next 200 frequent symbols is significantly large, almost twice the number of frequent sequences found in the test with a symbol set of 331 symbols (see Table 6). Nevertheless, the NLDFT algorithm performed significantly better than the other two approaches in all the tests, achieving a running time lower than the PLWAP algorithm's in two orders of magnitude and lower than the UDDAG algorithm's in one order of magnitude when the symbol set had 531 or more symbols. Finally, Table 6 shows the number of patterns found in each database used in this study case.

**Table 6:** Patterns per text database in the study case.

Database	Frequency Threshold	Patterns
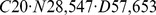	20	67,436
	18	88,852
	16	112,820
	14	161,871
	12	254,137
	10	528,737
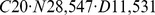 (20%)	10	32,823
D23,062 (40%)	10	102,582
D34,593 (60%)	10	135,029
D46,124 (80%)	10	255,674
D57,653 (100%)	10	528,737
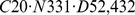	10	74399
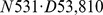	10	209754
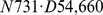	10	231354
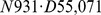	10	262561
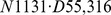	10	268484

Number of patterns obtained with each variation of the study case database and each frequency threshold used.

### Discussion

The experiments carried out showed that the proposed algorithm performs significantly better than the UDDAG and PLWAP algorithms, specially at lower supports. The experiments also showed how the processing time of the three algorithms increased as the minimum support was lowered, because this produces an increase in the number of patterns that meet the frequency threshold. Also, the proposed algorithm shows a better scalability than the UDDAG and PLWAP algorithms, as it maintains a good performance when increasing the number of sequences, the average sequence length or the size of the symbol set, while the UDDAG and PLWAP algorithms have a great decrease in performance when increasing the size of the dataset. Regarding memory efficiency, the NLDFT algorithm also performed better than the UDDAG and PLWAP algorithms. This is because the NLDFT algorithm, unlike the UDDAG and PLWAP algorithms, does not build a costly data structure holding the entire input database to perform the mining process, and neither stores candidate patterns to later test their frequency. The NLDFT algorithm only stores the necessary information for the pattern that is currently growing.

## Conclusions

Based on the experimental and theorical comparison with recent sequential pattern mining approaches, the NLDFT algorithm showed to be one of the most efficient for sequential pattern mining. The NLDFT algorithm has better peformance in running time and memory consumption than popular approaches. Additionaly, it has better scalability.

As future work, we will explore different approaches for improving the efficiency of the frequent symbol extraction process of the NLDFT algorithm, because this process is executed frequently. Approaches to solve the k-majority problem could be useful, since this problem is similar to the frequenty symbol extraction process. Among these approaches we will explore: using hash operations and linked lists to store the frequency counters [28] and using a parallel scheme, discovering local frequent symbols and then reducing that set to find the final set of frequent symbols [29]. Moreover, NLDFT has no restriction over the distance between the symbols of a frequent sequence (known as *gap restriction*), however, it would be desirable to give the user the option of setting a gap restriction as an input parameter of the algorithm, in order to obtain frequent sequences useful in areas such as Text Mining, where frequent sequences whose symbols are separated by long distances are not meaningful. A possible way to achieve this, is by assigning a restriction value to each growing frequent sequence, which would indicate the maximum distance where frequent symbols could appear after the sequence to be considered within the gap restriction.
